# Genome-wide association study of exotic *Fragaria* germplasm accessions for resistance to Phytophthora crown rot in strawberry

**DOI:** 10.1186/s12870-026-08186-6

**Published:** 2026-01-23

**Authors:** Mandeep Poudel, Anupam Gogoi, Jakob Junkers, Arne Stensvand, May Bente Brurberg, Jahn Davik

**Affiliations:** 1 Department of Plant Sciences, Norwegian University of Life Sciences, Ås, Norway; 2 Division of Biotechnology & Plant Health, Norwegian Institute of Bioeconomy Research, Ås, Norway

**Keywords:** Disease resistance, *Fragaria* × *ananassa*, *Phytophthora cactorum*, Genome-wide association study (GWAS), SNP marker

## Abstract

**Background:**

The soil-borne oomycete *Phytophthora cactorum* causes crown rot, a major disease of the allo-octoploid strawberry (*Fragaria × ananassa* Duch., 2n = 8× = 56) that limits cultivation worldwide. Resistance to *P. cactorum* is a highly desirable trait but is typically quantitative and moderately heritable. A better understanding of the genetic basis of resistance to crown rot is essential for developing durable crown rot-resistant cultivars.

**Results:**

We conducted a genome-wide association study (GWAS) using multi-locus models on 100 wild strawberry accessions from South and North America. The accessions were genotyped using the Axiom™ 50 K strawberry SNP array and mapped to the *F.* × *ananassa* cv. Royal Royce v. 1.0 reference genome. Testing for resistance to *P. cactorum* revealed a wide range of phenotypes. A single genetic marker, AX-184528282, located on chromosome 7B, was strongly associated with resistance to *P. cactorum* and explained 53% of the observed phenotypic variation. This marker was present in several highly resistant exotic *Fragaria* accessions that represent potential donors for introgression of favorable alleles into modern strawberry cultivars. In addition, several strong candidate resistance genes were identified within the 2 Mb genomic region surrounding the significant marker.

**Conclusions:**

This study advances understanding of resistance to *P. cactorum* in strawberry and identifies genetic resources that can accelerate the development of crown rot-resistant cultivars through marker-assisted breeding.

**Supplementary Information:**

The online version contains supplementary material available at 10.1186/s12870-026-08186-6.

## Introduction

The garden strawberry (*Fragaria × ananassa* Duch.) is an economically important soft fruit crop, valued for its appealing sensory qualities [1] and health benefits [2, 3]. Global production reached 10.5 million tonnes in 2023 [4] and has steadily increased over the years [5, 6]. However, commercial strawberry cultivars are vulnerable to several pathogens. Among these, the soil-borne hemibiotrophic oomycete *Phytophthora cactorum* (Lebert & Cohn) that causes leather rot of the fruit and crown rot is of particular concern, as it leads to substantial yield reduction and compromised fruit quality, resulting in considerable economic losses [7–10]. *Phytophthora cactorum* with its global distribution affects over 200 plant species [11]. As a homothallic pathogen, *P. cactorum* produces both sexual oospores, which can survive in soil for numerous years without their host plants, and asexual sporangia that release flagellated zoospores in the presence of water [12]; both sporangia and zoospores can infect the root and vascular tissues of plants [10, 13, 14].

The management of Phytophthora crown rot has become increasingly challenging. In the past, extensive reliance on effective soil fumigation using methyl bromide led to focus on fruit size and flavour in the breeding programs [15, 16]. Consequently, most cultivars developed after 1960 are susceptible to soil-borne pathogens like *P. cactorum* [16, 17]. Alternatives to methyl bromide have proven less effective [18–23]. The emergence of pesticide resistance in *P. cactorum* [15, 24, 25] has further undermined chemical control options.

*Fragaria × ananassa* originated in western Europe during the 1700s as a hybrid between *Fragaria chiloensis* (L.) Mill. and *Fragaria virginiana* Duch. The following 300 years of selection has reduced the genetic diversity and number of rare alleles in modern *F. × ananassa* breeding populations compared to the two progenitors [26, 27]. This reduction in diversity limits the adaptability to evolving threats such as *P. cactorum*. By contrast, *F. chiloensis* and *F. virginiana*, distributed across a broad range of habitats in the Americas, harbour extensive allelic diversity shaped by complex evolutionary histories and substantial interspecific gene flow [26–30]. *Fragaria virginiana*, commonly known as Scarlet strawberry, is native to inland habitats of North America and comprises four subspecies: ssp. *glauca*, ssp. *grayana*, ssp. *platypetala* and ssp. *virginiana* [31]. *Fragaria chiloensis*, also known as beach or Chilean strawberry, is native to the Pacific coastlines of both South and North America and Hawaii, with four subspecies: ssp. *chiloensis*, ssp. *lucida*, ssp. *pacifica* and ssp. *sandwicensis*. The three octoploid *Fragaria* species (*F. × ananassa*, *F. chiloensis*, and *F. virginiana*) can readily intercross, as there are no genetic barriers between them [32]. Thus, introgression of untapped alleles from these wild relatives into the garden strawberry offers a promising strategy for improving broad-spectrum resistance, particularly for complex, polygenic traits like resistance to *P. cactorum* [16, 17, 27].

Breeding for resistance to *P. cactorum* in the garden strawberry has traditionally relied on phenotypic selection. The allo-octoploid nature of *F. × ananassa* has posed challenges for developing high-throughput molecular markers and for mapping disease resistance loci. As a result, research has often focused on the less complex genome of the diploid *F.* vesca (2n = 2× = 14) [33–35], which represents one of the four diploid sub-genomes in *F*. *× ananassa* [36]. However, the recent release of chromosome-level genome assemblies for *F. × ananassa* cvs. Camarosa [36] and Royal Royce [37] along with the development of SNP genotyping platforms [32, 38, 39], has significantly enhanced the capacity to dissect the genetic architecture of disease resistance and other traits in the garden strawberry.

Genome-wide association studies (GWAS) offer a powerful tool to investigate natural variation and identify genetic loci associated with traits at a relatively high resolution. In strawberry, GWAS has successfully revealed QTLs and candidate genes linked to horticultural traits [40–43] as well as disease resistance [17, 33, 44–47]. In this study, GWAS was applied to a panel of 100 wild polyploid *Fragaria* accessions originating from different regions across the Americas to identify genetic loci associated with resistance to *P. cactorum*. A major-effect resistance-associated SNP and several candidate defence-related genes within a 2 Mb window surrounding this marker were identified. These findings provide insight into the genetic basis of Phytophthora crown rot resistance in wild *Fragaria* and offer valuable resources for resistance breeding in the garden strawberry.

## Materials and methods

### Strawberry plant material

Seed batches of 100 wild *Fragaria* accessions, originally identified as *F. chiloensis* (68 accessions) and *F. virginiana* (32 accessions), were obtained from the National Clonal Germplasm Repository, Corvallis, OR (https://www.ars-grin.gov/). Subsequent genetic analysis revealed several misclassifications: six accessions were identified as the decaploid (10×) species *F. cascadensis*, two as the naturally occurring hybrid *F.* × *ananassa* spp. *cuneifolia*, and one as an introgressed *F.* × *ananassa* [48]. After reclassification, the final set of accessions included *F.* × *ananassa* (3), *F. cascadensis* (6), *F. chiloensis* (67) and *F. virginiana* (24) (Supplementary File S1). The seeds were pretreated with concentrated H_2_SO_4_ for 12 min, rinsed thoroughly in an ice bath and dried. They were sown in nutrient rich compost soil (Øras Bed and potting soil, Mogreina, Norway) in greenhouse conditions with a 16 h photoperiod at 18 °C. After germination, one 3–4 week-old seedling from each of the 100 accessions was transplanted to a larger pot. Plants for the inoculation experiments were clonally propagated by runners from this single mother plant.

### *Phytophthora cactorum*

*Phytophthora cactorum* isolate 10,300 obtained from an infected rhizome of a field grown strawberry in Ås, Norway [49] was used for the inoculations. Low genetic variation, together with comparable aggressiveness, has been documented among strawberry crown rot isolates [34]. To produce sporangia, the isolate was cultured on 10% V8 agar plates (100 mL vegetable juice, 900 mL distilled water, 1 g CaCO_3_ and 15 g agar) at room temperature (~ 21 °C) in the dark for two weeks. Zoospore suspensions were prepared as described previously by Eikemo et al. [50], with slight modifications. Briefly, the *P. cactorum* agar plate cultures were covered with autoclaved MilliQ water, incubated at 4 °C for 1 h, and 30 min at room temperature to release the zoospores from the sporangia. The zoospores were counted using a hemacytometer, and the concentration was adjusted to 1 × 10^5^ spores/mL for inoculation. The zoospore suspension was prepared just before inoculation (maximum 1 h) to avoid the loss of viability of the zoospores.

### Disease resistance phenotyping

A total of 100 wild *Fragaria* accessions were tested for resistance to crown rot using a modified block design in five independent greenhouse trials conducted at the Centre for Plant Research in Controlled Climate, Ås, Norway, from December 2022 to October 2023. Due to variation in the availability of clonal plant material among trials, not all accessions were included in each trial; consequently, the trials were analysed as a connected set based on overlapping genotypes. For each accession, three to five clonal plants (3–4 weeks old) were tested per trial. Plants were lightly watered 2–3 h before inoculation. The base of the rhizomes (crowns) was gently scraped with a sterile scalpel just before inoculating with 2 mL of zoospore suspension (1 × 10^5^ spores/mL). To minimize any loss of inoculum, overhead irrigation was avoided during the first week after inoculation. Plants of the crown rot susceptible cv. Polka [51] were included as a positive control to provide a qualitative check of inoculum viability and the effectiveness of the inoculation procedure.

The severity of the disease symptoms was visually monitored once per week over a 4-week period and scored on a scale from 1 to 8 according to Bell et al. [52]. Plants that died within the first, second, third or fourth week after inoculation were scored 8,7,6 or 5, respectively. The rhizomes of plants that survived this period were dissected longitudinally to assess the degree of necrosis and scored using the following scale: 4 - severe necrosis covering more than 50% of the rhizome area; 3 - small patches of necrotic lesions; 2 - minor brown speckles; 1 - no visible symptoms in the rhizome.

### Phenotypic data analysis

Analysis of variance (ANOVA) of the raw phenotypic scores was performed using a linear mixed model (LMM) fitted with the lme4::lmer() function in R [53]. All factors including accession, trial and their interaction were treated as random effects. Adjusted phenotypic means for each accession were estimated based on the following model:

$$Y_{ijk}=\mu+R_i+G_k+{\left(RG\right)}_{ik}+\varepsilon_{ijk}$$

where *Y*_*ijk*_​ is the phenotype for the *j*^*th*^ clonal plant of the *k*^*th*^ accession in the *i*^*th*^ trial, *µ* is the overall mean, *R*_*i​*_ is the random effect of the *i*^*th*^ trial, *G*_*k​*_ is the random effect of the *k*^*th*^ accession, (*RG*)_*ik*_​ is the random interaction effect between trial and accession, and ϵ_*ijk*_​ is the residual error. The final adjusted means (= disease scores) for each accession obtained from this model were subsequently used for further analyses and data visualization. Resistance categories were defined using k-means clustering of adjusted-mean disease scores for each accession. Broad sense heritability (*H*^*2*^) was estimated using the “bwardr::Cullis_H2” function in R [54], following the method described by Cullis et al. [55]. The normality of disease scores was assessed using Quantile-Quantile (Q-Q) plot, histogram, and Kurtosis test.

### SNP genotyping and marker distribution

Newly emerged, non-expanded leaf tissues were harvested from the greenhouse grown seedlings. Fifty milligrams of leaf tissue from each ecotype was freeze-dried in Corning^®^ 96 well PP 1.2 mL cluster tubes (Sigma-Aldrich, Germany). The SNP genotyping was performed by the TraitGenetics Section at SGS Institut Fresenius GmbH, Gatersleben, Germany using the Axiom™ Strawberry FanaSNP 50 K array [32] from Affymetrix^®^ Inc., Santa Clara, California, USA. The amenable class of markers from the Poly High Resolution (PHR) and No Minor Homozygote (NMH) categories were selected from the default classes generated by the Affymetrix software SNPolisher. The SNPs were coded as “0” (homozygous for the reference allele), “1” (heterozygous) and “2” (homozygous for the alternative allele). Quality control (QC) and imputation of missing SNPs were performed using the R package *snpReady* [56]. The markers with a call rate below 90% and a minor allele frequency (MAF) lower than 0.05 were removed. For imputation of missing SNPs, Wright’s equilibrium method [57] was used, which estimates allele occurrence based on the observed allelic frequency and the heterozygosity of existing markers. After eliminating non-informative markers and those with no known positions in the ‘Royal Royce’ reference genome [37], a total of 15,633 markers with high-quality bi-allelic clusters were selected for the subsequent GWAS analysis. For visualising distribution of markers, a SNP marker density plot was generated using the SRplot web tool [58].

### Population structure analysis

A dataset of 2,849 uncorrelated SNPs was obtained by pruning adjacent SNP markers with a linkage disequilibrium (LD) threshold of 0.2 using the *SNPRelate* R package [59]. This LD-pruning step ensured that the SNPs used for population structure analysis were statistically uncorrelated, minimising bias from physical linkage. The number of sub-populations among the *Fragaria* accessions was then inferred using the LD-pruned SNP dataset in STRUCTURE v.2.3.4 software [60]. To determine the optimal number of sub-populations, the simulation was run with a burn-in period of 10,000 iterations, followed by 50,000 Markov Chain Monte Carlo (MCMC) replications. This procedure was repeated across 10 iterations for each potential number of clusters (*K* = 1 to 6). Default parameters were used, except for the initial alpha (α), which was adjusted as recommended by Wang [61], to avoid inaccurate cluster estimation caused by unbalanced sampling. The optimal number of clusters (*K*) was estimated using both STRUCTURE HARVESTER v0.7 [62] with the ∆*K* method [63] and KFinderV1.0, applying the parsimony method [64]. The resulting matrices indicating the optimal *K* and the corresponding structure barplots were generated using the *ggplot2* [65] and the pophelperShiny v2.1.1 [66], R packages respectively.

The population structure was further analysed using a principal component analysis (PCA) using the base R function *prcomp*() and plotted with *ggplot2* [65]. Additionally, a marker based kinship matrix (*K*) was generated using the VanRaden kinship algorithm [67] in GAPIT (Genome Association and Prediction Integrated Tool) v3.4.0 [68]. The heatmap from the kinship matrix was visualised using the *pheatmap* R package [69].

### Genome-wide association study

To investigate the genetic factors associated with resistance to *P. cactorum* in the wild *Fragaria* accessions, a GWAS was conducted using adjusted mean disease scores and 15,633 quality controlled SNP markers, as inputs to the R package GAPIT v3.4.0 [68]. Two multi-locus statistical models; BLINK (Bayesian-information and Linkage-disequilibrium Iteratively Nested Keyway) [70] and MLMM (Multiple Loci Mixed Model) [71] were used to identify the marker-trait associations (MTAs). The multi-locus analyses incorporate multiple markers simultaneously as co-variates while reducing the false discovery rate [68, 72]. The information from the PCA analysis including the first two principal components and the *K* matrix was used as covariates to control for confounding effects in GAPIT. The Bonferroni correction (0.05/ number of markers) with *p*-value threshold of -log_10_(*p*) = 5.49 was used to identify significant MTAs. To illustrate the genomic association results, Manhattan plots and Q-Q plots were generated. The proportion of phenotypic variation explained (PVE) by the significant marker was estimated using the “random.model = TRUE” setting in the GAPIT package [68], which fits each significant marker as a random effect in a linear mixed model.

### Candidate gene identification underlying crown rot resistance

To locate candidate genes around the significant SNP, local LD block was constructed using the LDBlockShow v1.40 software [73]. Furthermore, the linkage disequilibrium among SNP marker pairs was calculated using TASSEL v5.0 [74]. A non-linear regression model was implemented using a custom R script to calculate the intra-chromosomal LD decay distance at which the correlation decreased to half of its maximum value. The results from the LD decay distance and LD block analyses were considered to predict a genomic region of ± 1 Mb around a significant SNP at chromosome 7B. This genomic region was used to search for candidate genes in the Royal Royce v. 1.0 reference genome (https://phytozome-next.jgi.doe.gov/info/FxananassaRoyalRoyce_v1_0). The function of each gene transcript was retrieved using the OmicsBox Blast2GO annotation tool.

## Results

### Disease resistance in wild *Fragaria* accessions

**Fig. 1:**
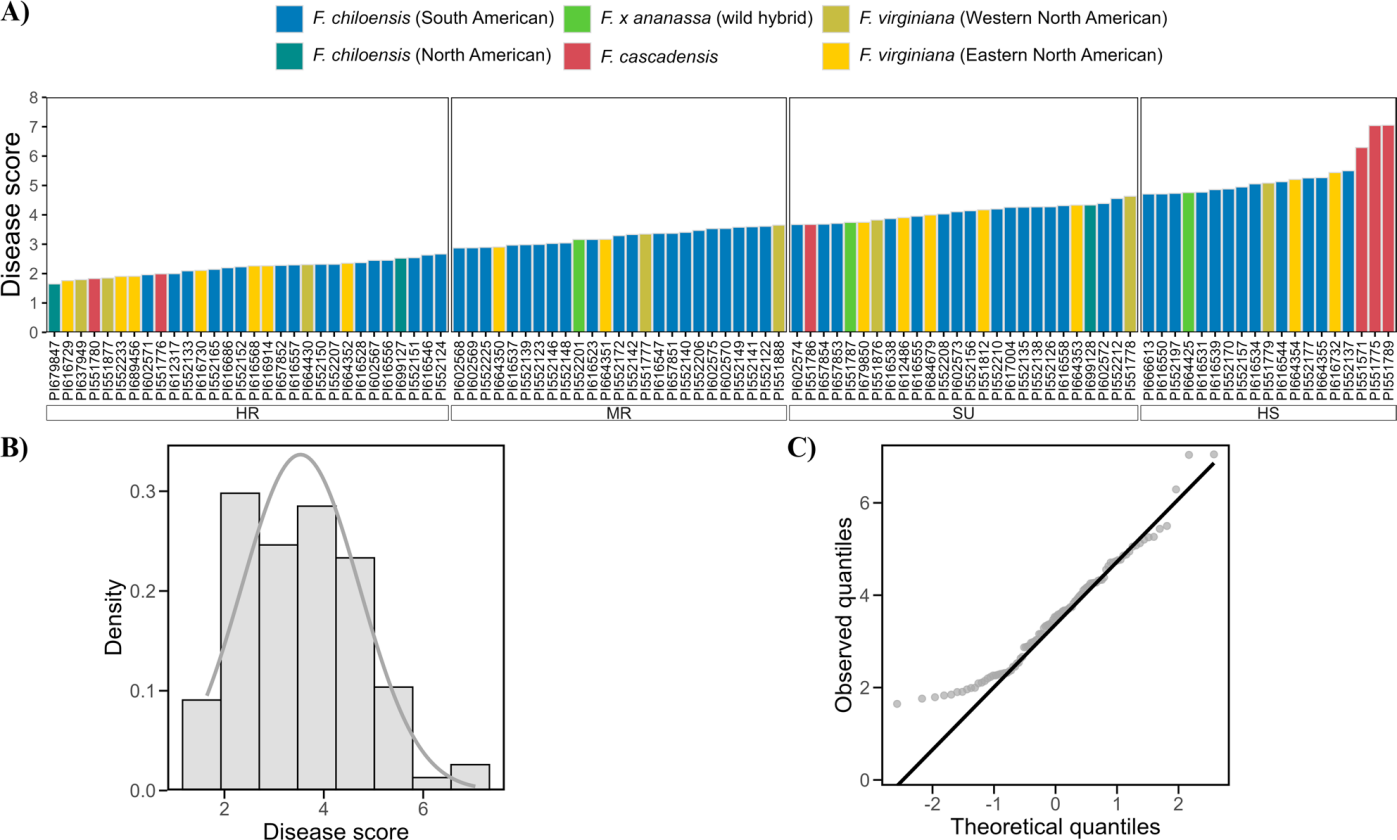
Distribution of disease scores for resistance to Phytophthora crown rot among 100 wild *Fragaria* accessions. **A** Disease scores (adjusted means from the mixed-effects model) across genotypes, with *Fragaria* species (colour coded bars). Resistance levels are classified as highly resistant (HR: < 2.70), moderately resistant (MR: 2.70 to < 3.66), susceptible (SU: 3.66 to < 4.63), and highly susceptible (HS: > 4.63). **B** Histogram of observed disease score densities with a fitted normal distribution curve (grey line). **C** Quantile-Quantile (Q-Q) plot comparing the observed quantiles with the theoretical normal distribution

The resistance to crown rot varied substantially between different accessions of the wild *Fragaria* (Supplementary File S1). Resistant phenotypes were observed across different *Fragaria* species and subspecies, with variability detected both among and within species from different geographical origins. The accessions were classified into four categories using k-means clustering (k = 4) on adjusted mean disease scores: highly resistant (< 2.70), moderately resistant (2.70 to < 3.66), susceptible (3.66 to < 4.63) and highly susceptible (> 4.63) (Fig. 1A). Among the 100 tested accessions, the three most resistant were *F. chiloensis* ssp. *pacifica* (PI679847), *F. virginiana* ssp. *virginiana* (PI616729) and *F. virginiana* ssp. *platypetala* (PI637949), with adjusted mean disease scores of 1.64, 1.75 and 1.79, respectively. The accession PI679847 from *F. chiloensis* ssp. *pacifica* showed no disease symptom across multiple replications. Additionally, 34 accessions were moderately resistant, exhibiting mild necrotic symptoms (Fig. 1A). In contrast, 63 accessions of different species developed severe crown rot symptoms. Among these, 11 showed high mortality and were categorised as highly susceptible (Fig. 1A). Notably, the three most susceptible accessions (PI551789, PI551775, PI551571) were *F. cascadensis* and showed complete collapse within the first two weeks. The adjusted mean disease scores ranged from highly resistant to highly susceptible with a modest peak (kurtosis = 3.1) and slight skewness towards resistance (skewness = − 0.5; Fig. 1B and C; Fig. S1). The linear mixed model revealed that all variance components contributed significantly to the observed variation (Table 1). The broad sense heritability was high, with an estimated H^2^ = 0.77 (Table 1).

**Table 1:** Descriptive statistics and broad-sense heritability (H^2^) of resistance to Phytophthora crown rot in wild *Fragaria* accessions

*N* ^a^	Mean ^b^	SD ^c^	CV ^d^	Accession	Trial	Accession × Trial	H^2 e^
1310	3.53	2.49	0.70	101.9***	37.8***	38.5***	0.77

^a^Number of observations

^b^Adjusted mean (= disease score) estimated from the linear mixed model

^c^Standard deviation based on model-estimated variance

^d^Coefficient of variation

^e^Broad-sense heritability

***p < 0.001

### Genome-wide distribution of SNP markers

**Fig. 2:**
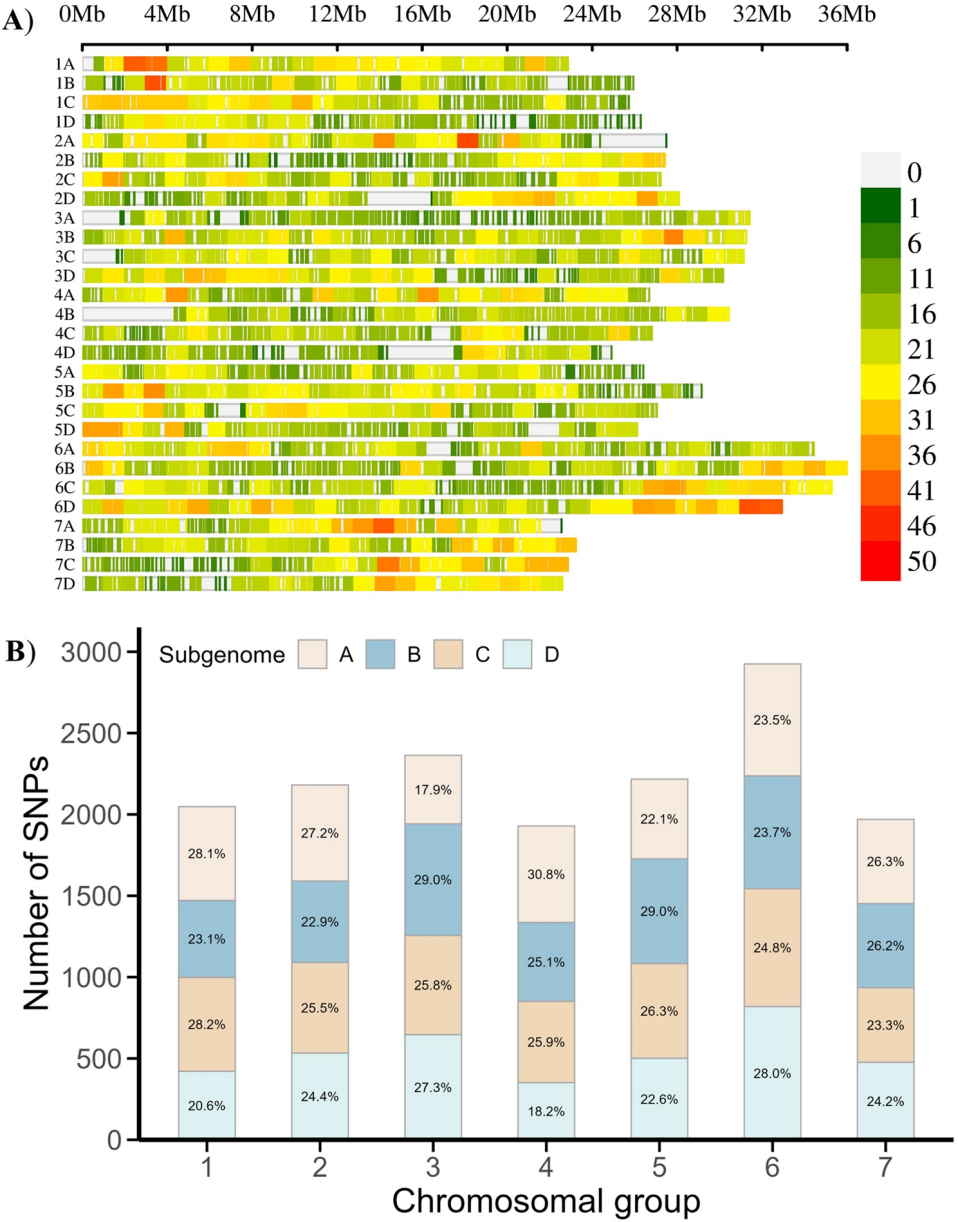
Distribution of 15,633 SNPs from 100 wild *Fragaria* accessions across the sub-genomes of the *F.* × *ananassa* cv. Royal Royce v. 1.0 reference genome. **A** Density plot of SNP markers in the 28 chromosomes. The colour key represents number of markers within a 1 Mb window size; the horizontal axis shows the chromosome length (Mb). **B** Distribution of SNPs on seven chromosomal groups and the percentage of mapped SNPs on the respective sub-genomes A, B, C and D

A total of 49,483 markers were obtained from the 50 K Fana SNP array, of which the majority (64%) were classified as either poly-high resolution (PHR; 27,721) or no-minor homozygote (NMH; 3,974) polymorphisms (Table S1). These 31,695 high-confidence SNPs were retained for further filtering to remove markers lacking a defined genomic position in the reference assembly. Subsequent quality control steps excluded markers with more than 10% missing data and those with a minor allele frequency (MAF) below 5%, resulting in a final set of 15,633 high-quality biallelic SNP markers **(**Supplementary File S2). Physical mapping showed that these markers were evenly distributed across all chromosomes and sub-genomes, with an average of 558 markers per chromosome (Fig. 2; Table S2). This even distribution reduces the likelihood of missing important genomic regions. Chromosome 4D (24.9 Mb) contained the lowest number of markers (351), whereas chromosome 6D (32.9 Mb) had the highest number (819). The overall average marker density was approximately one SNP per 51 kb. The highest marker density was observed on chromosome 1A, with one marker every 39.6 kb, whereas chromosome 3A had the lowest density, with one marker per 74.6 kb.

### Population structure and genotypic relationships

**Fig. 3:**
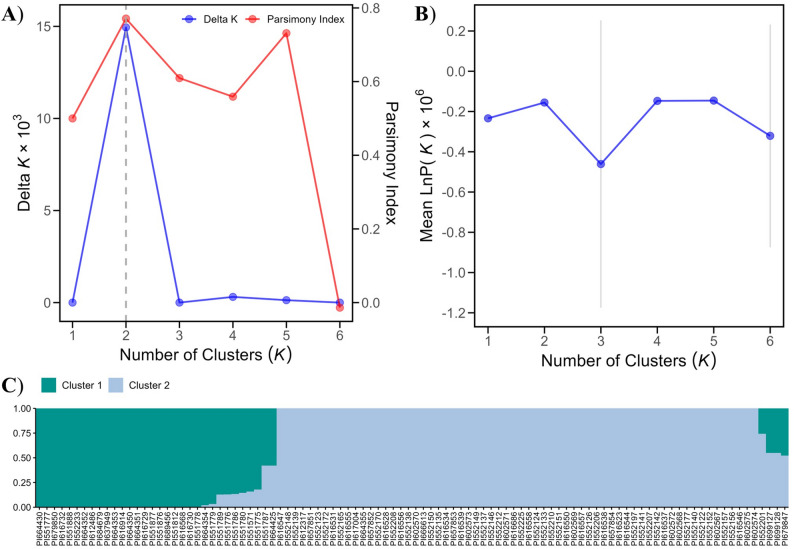
Population structure analysis of 100 wild *Fragaria* accessions based on 2,849 linkage disequilibrium (LD) pruned SNP markers. **A** Optimal number of clusters (*K* = 2), based on the Δ*K* method and parsimony index, is indicated by the vertical dashed line. **B** Mean log probability [LnP(*K*)] and its standard deviation for each tested *K* value. **C** STRUCTURE bar plot showing the proportion of ancestry for each individual in the two inferred genetic clusters; each vertical bar represents an accession, with colours indicating cluster membership

**Fig. 4:**
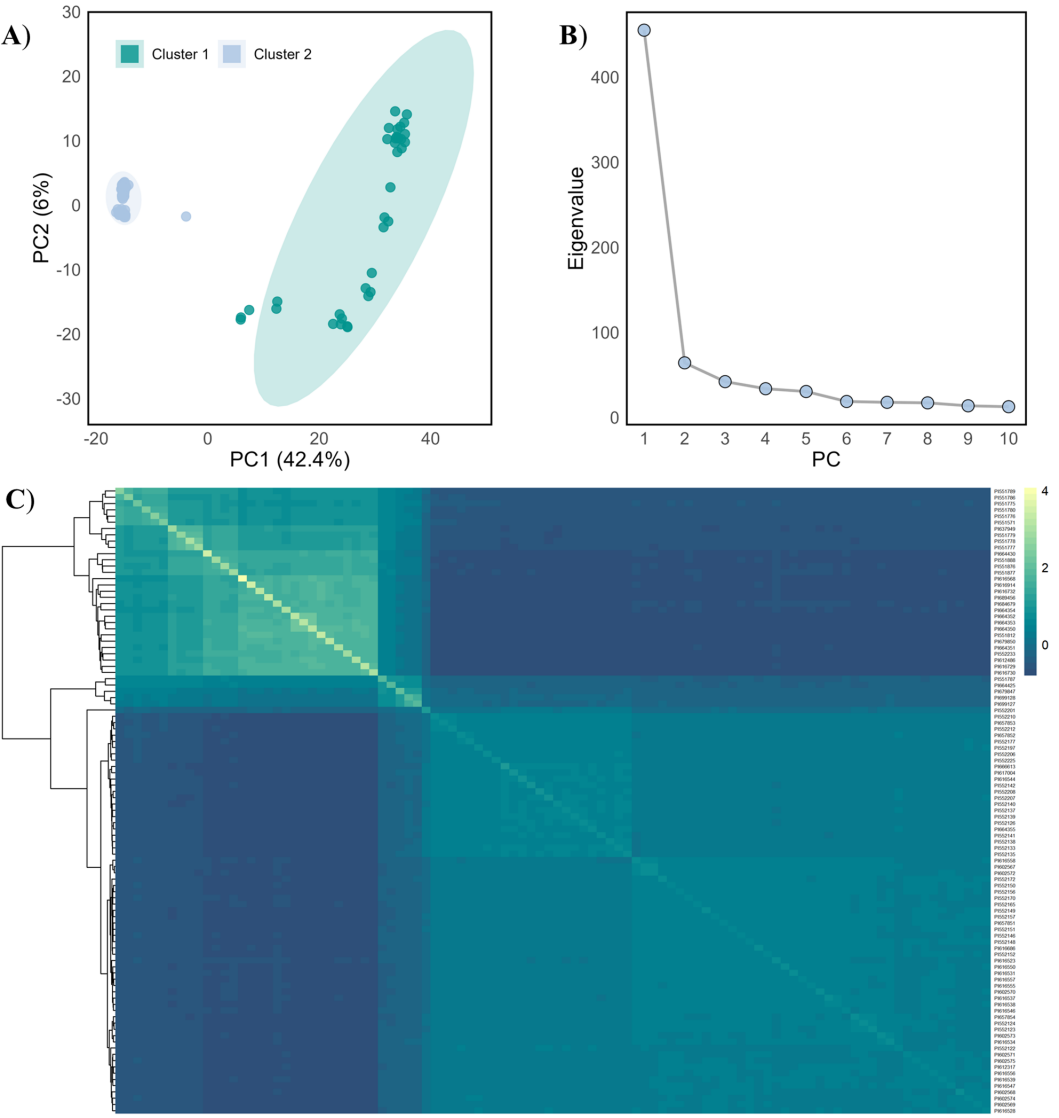
Genetic relationship among 100 wild *Fragaria* accessions based on 2,849 linkage disequilibrium (LD) pruned SNP markers. **A** Principal component analysis (PCA) plot based on the first two principal components; each point represents a single accession. The first two PCs together explained approximately 48% of the total genetic variation. **B** Scree plot showing the eigenvalues of the first ten principal components; the first two PCs, identified using the elbow method, were selected for the GWAS analysis. **C** Heatmap of the kinship matrix, illustrating the degree of relatedness among the wild *Fragaria* accessions. The scale represents pairwise kinship coefficients, where higher values indicate greater genetic relatedness between accessions

The population structure of the wild accessions was assessed using a dataset of 2,849 LD-pruned SNP markers employing STRUCTURE, principal component analysis (PCA), kinship estimation, and neighbor-joining (NJ) clustering approaches. The most likely number of clusters among the studied *Fragaria* accessions was two (*K* = 2), supported by both ΔK values and the highest parsimony index (Fig. 3A and B). Cluster 1 predominantly comprised *F. virginiana* accessions, and cluster 2 contained *F. chiloensis* accessions, with a few admixed individuals observed between the clusters (Fig. 3C). The PCA result confirmed this pattern, separating the accessions into two distinct clusters, with the first two principal components, PC1 (42.4%) and PC2 (6.0%), explaining much of the genetic variation (Fig. 4A and B). The kinship matrix heatmap (Fig. 4C) and the NJ-phylogenetic tree (Fig. S2) also indicated a similar clustering pattern.

### Genome-wide association analysis

**Fig. 5:**
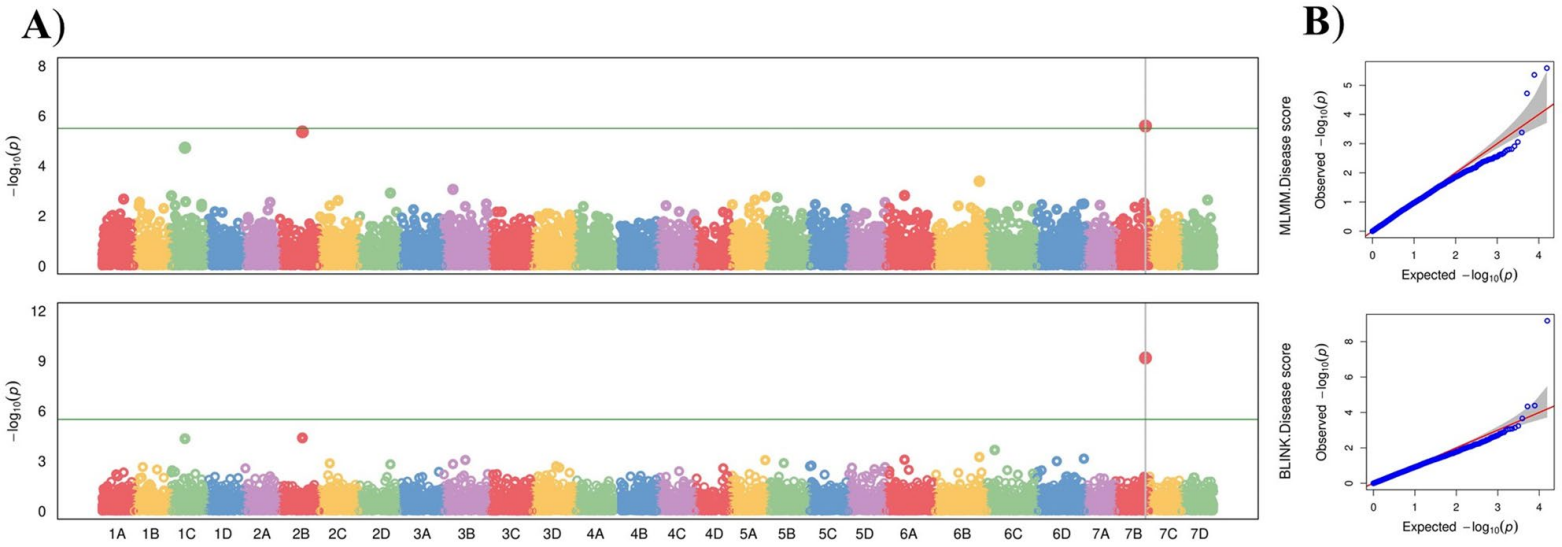
Genome wide association study for resistance to Phytophthora crown rot in 100 wild *Fragaria* accessions with 15,633 SNP markers. **A** Stacked Manhattan plots from BLINK and MML GWAS models. Each dot represents a SNP; the X-axes show the 28 chromosomes of octoploid strawberry, and the Y-axes show the –log_10_(p) value for each SNP. The Bonferroni significance threshold of –log_10_(p) = 5.49 is indicated by horizontal green lines, and the SNP exceeding this threshold is indicated by a vertical grey line. **B** The corresponding Quantile-Quantile (Q-Q) plots for each model, comparing the expected (red line) and observed (blue dots) –log_10_(p) values. The grey shaded areas represent the 95% confidence interval, illustrating deviation observed from the expected distribution

A genome-wide association study (GWAS) for resistance to Phytophthora crown rot in wild *Fragaria* accessions was performed using 15,633 high-quality biallelic SNP markers and two multi-locus models, BLINK and MLMM. Both models consistently identified a single significant marker (AX-184528282) located near the distal end of chromosome 7B at position 21,046,884 to be significantly associated with crown rot resistance (Fig. 5A; Table 2). Deviation of the observed LOD scores (-log_10_(*p*)) from the expected distribution in the upper-tail of the Q-Q plot further supported the adequacy of both models (Fig. 5B). The marker AX-184528282 explained 53.3% of the phenotypic variance for crown rot resistance, with a minor allele frequency (MAF) of 0.15 and an estimated allelic effect of -1.03 to -1.18 (Table 2).

**Table 2:** Genome-wide association study (GWAS) summary of the SNPs associated with resistance to Phytophthora crown rot in *Fragaria* accessions

SNP	Chr ^b^	Position	MAF ^c^	GWAS model	−log10(*p*)	Effect	PVE (%) ^d^
AX-184528282 ª	7B	21,046,884	0.14	BLINK	9.18	-1.18	53.3
				MLMM	5.59	-1.03	53.3
AX-184092248	2B	15,724,363	0.19	BLINK	4.38	0.70	
				MLMM	5.36	0.94	
AX-184840855	1C	9,781,877	0.49	BLINK	4.34	-0.72	
				MLMM	4.72	-0.79	

ªSignificant SNP detected by both GWAS models

^b^Chromosome number

^c^Minor allele frequency (MAF)

^d^Phenotypic variance explained (PVE)

**Fig. 6:**
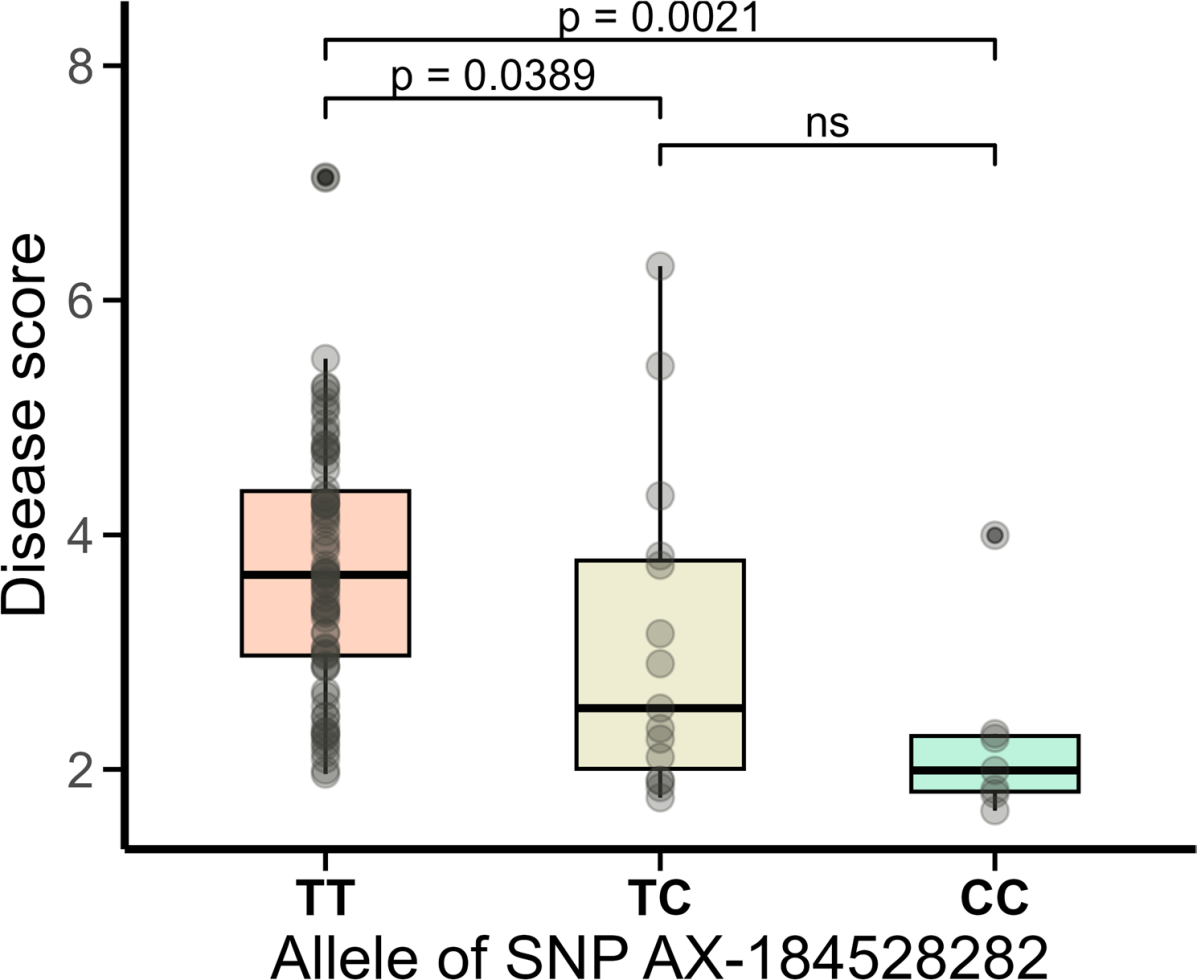
Box plot showing the allele effect of the associated T/C single nucleotide polymorphism (SNP) AX-184528282 on Phytophthora crown rot severity in 100 wild *Fragaria* accessions. The C allele is associated with lower disease severity (favourable), while the T is associated with higher disease severity (unfavourable). Each dot represents an individual genotype. *p*-values from pairwise comparisons between groups, assessed using Dunn’s post-hoc test with Benjamin-Hochberg adjustment, are displayed on the top of the plot; “ns” indicates non-significant

A Kruskal-Wallis test showed that a T/C base transition at this locus was significantly associated with reduced susceptibility to Phytophthora crown rot (*p* = 0.00057). Pairwise comparisons using Dunn’s test with Benjamin-Hochberg adjustment revealed significant differences in disease scores between TT and CC homozygotes and between TT and TC. No significant difference was observed between TC and CC (Fig. 6). The CC homozygotes (*n* = 7) showed a markedly lower median disease score (1.99) compared to TC heterozygotes (2.52; *n* = 15) and TT homozygotes (3.66; *n* = 78) (Fig. 6). In addition to the significant association detected on chromosome 7B, two markers close to the significance threshold (-log_10_(*p*) = 5.49) were identified on chromosome 1C (AX-184840855 at position 9,781,877) and chromosome 2B (AX-184092248 at position 15,724,363) (Table 2; Fig. 5B).

### Potential candidate genes involved in crown rot resistance

**Fig. 7:**
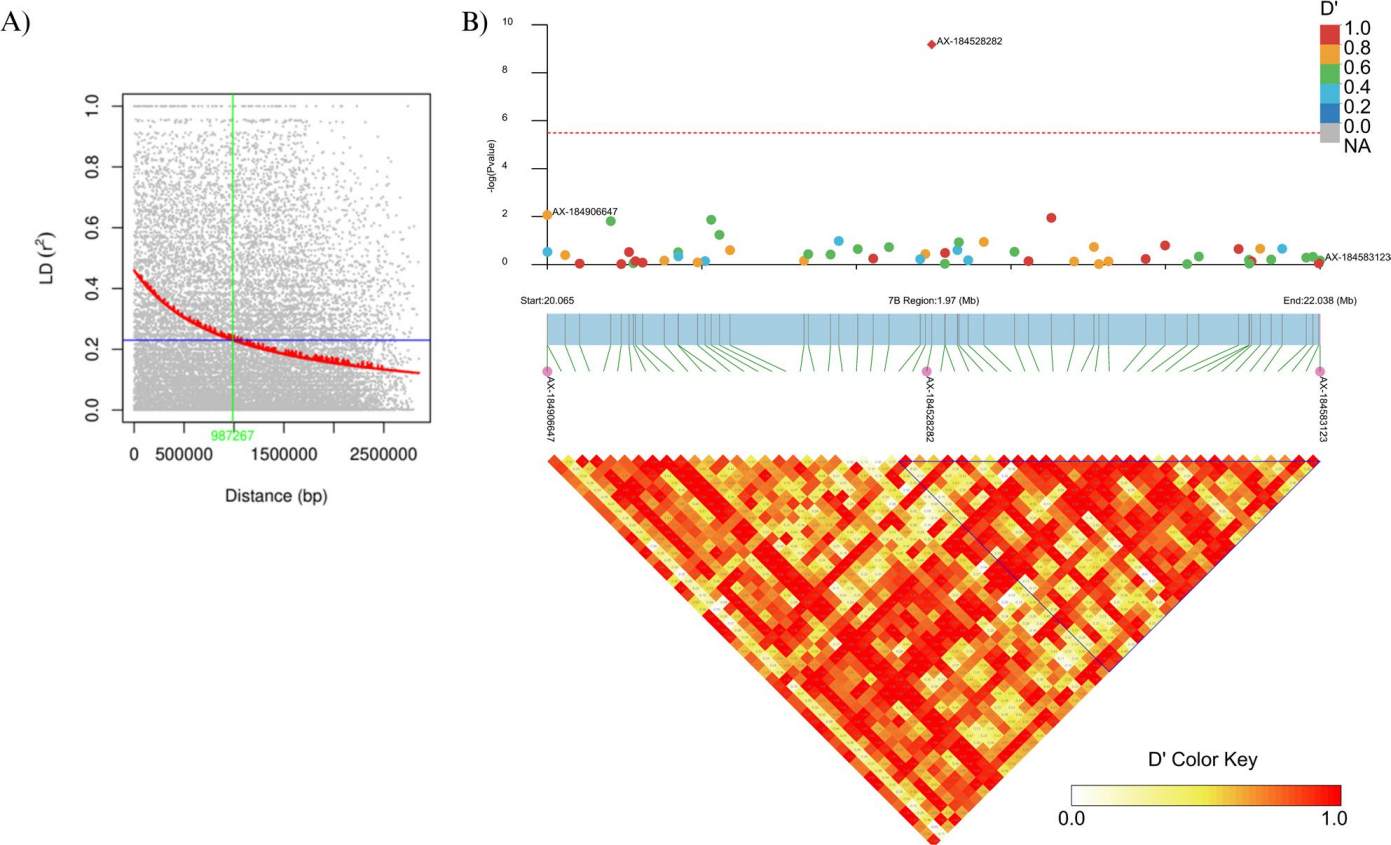
Linkage disequilibrium (LD) decay and LD heatmap for wild *Fragaria* accessions on chromosome 7B. **A** LD decay plot showing the decline in LD (r^2^) with increasing physical distance between intra-chromosomal marker pairs on chromosome 7B. The red trendline, represents the nonlinear regression fit; the horizontal blue line indicates the half LD decay threshold, and the vertical green line marks the corresponding physical distance at which this threshold is reached. **B **Top panel: Enlarged Manhattan plot highlighting a 2 Mb region containing 56 SNPs including the significant marker AX-184528282 (red diamond), which is associated with resistance to Phytophthora crown rot. The horizontal red dashed line marks the genome-wide significant threshold at -log_10_(*p*) = 5.49. Bottom panel: LD heatmap displaying pairwise LD (D’) among the 56 SNPs in the region. The pink dots indicate the boundaries of the 2 Mb region, with AX-184528282 centred. The highlighted triangle indicates a linkage block of 26 SNPs spanning an 896 kb region, with AX-184528282 positioned at the leftmost end of the block. The D’ colour key represents the strength of linkage disequilibrium, where darker colours indicate stronger LD

To identify candidate genes potentially contributing to *Phytophthora* crown rot resistance, the local linkage disequilibrium (LD) decay was analysed, and the LD block surrounding the significant marker was visualized. The average distance at which LD decayed to half its maximum value (0.23 from an initial 0.47) was estimated at 1.75 Mb (Fig. S3). The extent of LD decay varied among chromosomes, with Chromosome 7B showing the half-decay distance of 0.99 Mb (Fig. 7A; Table S2). A heatmap of pairwise LD revealed an 896 kb linkage block including the significant marker (Fig. 7B).

Based on the LD decay pattern and the identified linkage block on chromosome 7B, a ± 1 Mb genomic region, surrounding the significant SNP (AX-184528282), was investigated for potential disease resistance genes. Within this region, a total of 367 genes comprising 495 transcripts were retrieved from the Phytozome database (*F. × ananassa* cv. Royal Royce v. 1.0) (Supplementary File S3). BLAST2GO annotation identified 104 genes with motifs or predicted functions associated with plant immunity. Additionally, Gene Ontology (GO) enrichment analysis indicated that 124 genes were associated with the GO biological process “response to stress” (Fig. S4).

Through annotation and manual curation, several putative defence associated genes were identified, including those encoding serine/threonine kinases, receptor-like protein kinases (RLKs), nucleotide-binding leucine-rich repeat proteins (NLRs) including CNLs (coiled-coil (CC)-NLRs) and TNLs (Toll/interleukin-1 receptor (TIR)-NLRs), ABC transporter ATP-binding proteins, F-box domain-containing proteins, immunity associated WRKY transcription factors, Cytochrome P450 family proteins, Ca^2+^ dependent protein kinases (CDPKs), cyclic-nucleotide-gated calcium channels (CNGCs), cell wall modification enzymes (e.g., O-acetyltransferases, pectinacetylesterase inhibitors, pectinesterases), mitogen-activated protein kinases (MAPKs) and various zinc finger proteins (A20/AN1/C2H2/CCG/RING-type) (Supplementary File S3).

## Discussion

In this study, we investigated natural genetic variation in wild *Fragaria* accessions to uncover potential sources of resistance to *Phytophthora* crown rot. Artificial inoculation with *P. cactorum* revealed substantial variation in resistance among the accessions tested. The wide range of phenotypes, from highly resistant to highly susceptible, both within and among *Fragaria* species suggests that resistance to *P. cactorum* is not restricted to a particular species or subspecies. Similar distributions of resistant phenotypes among octoploid progenitors have been reported previously [17, 75]. Notably, among the 100 wild accessions evaluated, PI679847 was the only genotype that consistently exhibited complete resistance, showing no symptom development across repeated trials. This accession was the sole representative of *F. chiloensis* spp. *pacifica* in our panel. Interestingly, *F. chiloensis* spp. *pacifica* as well as a cultivar derived from it were among the most resistant accessions in a study of another soil-borne pathogen, *Verticillium dahlia* [76]. These findings highlight the potential of *F. chiloensis* spp. *pacifica*, native to the Pacific coast of northwestern North America, as a valuable genetic resource for introducing novel resistance alleles into modern strawberry cultivars.

Six accessions included in this study were originally classified as *F. virginiana* but were reclassified as *F. cascadensis* by Junkers [48] during the period in which this study was conducted. Collected from the Cascade Mountains, these accessions represent the first set of *F. cascadensis* evaluated for disease resistance, although anthocyanin content [77] and volatile compounds [78] have been previously analysed. These accessions showed a broad distribution of resistance responses: two were among the most resistant, one had an intermediate score, and three were the most susceptible accessions in the study.

The mixed linear model used in this study revealed that all variance components contributed significantly to explaining the variability in disease scores, indicating that both environmental factors and individual genetic backgrounds influenced the observed phenotypic responses. A broad-sense heritability (H^2^) of 0.77 for resistance to *P. cactorum* was estimated for the wild *Fragaria* accessions. This is consistent with the findings of Jimenez et al. [17], who reported similar heritability values (H^2^ = 0.75–0.81) for exotic ecotypes of octoploid *Fragaria* species. The high heritability of resistance to crown rot indicates that a substantial proportion of the phenotypic variation is genetically determined. This suggests good potential for selecting and transferring novel resistance alleles from phylogenetically diverse *Fragaria* species into modern cultivars. Such introgression could help broaden the genetic base of cultivated strawberries, the majority of which currently lack effective resistance to *P. cactorum* [17].

The results from the population structure analyses confirmed the known differentiation of the reference population according to geographical origin. Consistent with previous investigations [26, 79], the three North American *F. chiloensis* (ssp. *pacifica* and ssp. *lucida*) accessions clustered together and were clearly distinct from their South American counterpart, *F. chiloensis* ssp. *chiloensis*. Moreover, all the South American *F. chiloensis* accessions formed a well-defined cluster with minimal admixture, as expected given their monophyletic origin [28] and relatively lower mutation rates [26]. *Fragaria virginiana* accessions were also genetically distinct and exhibited subspecies-specific clustering, while all six *F. cascadensis* accessions formed a separate group positioned closer to the *F. virginiana* cluster. Overall, clear genetic differentiation among individuals of the different *Fragaria* species was evident in the present investigation.

We identified a single major effect SNP marker (AX-184528282) on Chromosome 7B associated with Phytophthora crown rot resistance in the wild *Fragaria* accessions. This SNP explained slightly more than half of the phenotypic variance among the tested *Fragaria* genotypes. Several QTLs and markers linked to Phytophthora crown rot resistance have been identified in strawberry [17, 35]. For instance, the major resistance locus *RPc-1*, mapped to *Fragaria vesca* LG6, explained 74.4% of the observed phenotypic variance [35]. Another, large effect QTL, *RPc2*, explained 13.7 to 25.3% of the phenotypic variation in *Fragaria × ananassa* [80]. Recently, a locus associated with *RPc2* on chromosome 7B was reported to explain 33.6 to 43.4% of the phenotypic variance [17]. The SNP marker (AX-184528282) identified in our study is located approximately 1.29 Mb upstream of the marker identified by Jimenez et al. [17] and approximately 1.09 Mb upstream from the left border of the fine-mapped *RPc2* locus [81]. The physical proximity of these markers at the distal end of chromosome 7B suggests that they are linked to the same Phytophthora crown rot resistance locus or gene cluster. This proximity, together with comparisons to previous studies, strengthens the evidence that AX-184528282 tags a conserved resistance region. Future work, including segregation analysis and fine-mapping, will be needed to confirm whether these nearby loci represent the same causal variant.

Using marker-based association analyses, LD decay and LDBlockShow, a 2 Mb genomic region strongly associated with Phytophthora crown rot resistance was identified. This region spans 1 Mb upstream and downstream of the SNP marker AX-184528282. Within this region, a total of 104 genes potentially associated with *P. cactorum* defence were annotated. The candidate most proximal to AX-184528282, *F×a7Bg202733* (21042724–21046636 bp) encodes a homeodomain leucine-zipper (HD-Zip) transcription factor (TF). This protein features a DNA-binding homeobox domain at the N-terminal and a central START (steroidogenic acute regulatory protein-related lipid transfer) domain [82, 83]. Several HD-Zip TFs have been reported for their involvement in biotic and abiotic stress responses [84–89]. For instance, overexpression of the HD-Zip gene *CaHB1* enhanced resistance to *Phytophthora infestans* in tomato by promoting the thickening of cell walls and the cuticle layer [85]. Additionally, HD-Zip TFs have been implicated in root development, phospholipid signalling in the roots and the regulation of vascular activities [90], thereby indicating their potential in enhancing resistance to biotic stress [91].

Genes encoding proteins related to Ca^2+^ signalling were identified within the candidate region of chromosome 7B, including a CNGC homolog and two Ca^2+^ sensor homologs: a relay protein CBL (calcineurin B-like protein) and a responder protein CDPK. These proteins are candidates for mediating early immune responses by facilitating PAMP (pathogen-associated molecular pattern)-triggered Ca^2+^ fluxes, where CNGCs provides the Ca^2+^ channelling pathway that can activate CBL and CDPK signalling cascades [92]. These findings align with recent studies that underscore the potential role of CNGCs in Phytophthora crown rot resistance, as demonstrated by GWAS analyses [17, 81].

Another notable category of genes potentially involved in defence in this region includes three genes encoding NLRs (*F×a7Bg202617*, *F×a7Bg202642* and *F×a7Bg202672*) and eight genes encoding RLKs. The NLRs are the largest class of resistance proteins in plants [93, 94] and these are known to confer resistance to *Phytophthora* spp. by recognizing pathogen RxLR effectors [95]. Notably, the octoploid *F. × ananassa* genome encodes 975 NLRs [94]. Among these, Fxa7Bg202672, a TNL-class NLR, has an integrated WRKY domain (with the conserved WRKYGQK motif) at its C-terminus. Such non-canonical integrated domains can act as decoys for pathogen effectors while mediating transcriptional regulation [96–98]. In addition to their effector recognition function, TNL-WRKY proteins have been found to work in pairs with other TNLs and confer resistance to a broad range of pathogens across various plant taxa [99, 100]. Furthermore, four genes homologous to pectin methylesterase inhibitor genes that are associated with cell wall reinforcement and enhanced resistance to fungal pathogens [101, 102], were also identified in this region. Taken together, these putative genes are strong candidates for further investigation of their roles in resistance to Phytophthora crown rot.

In addition to the significant SNP marker identified on chromosome 7B, two additional SNPs - AX-184840855 on chromosome 1C and AX-184092248 on chromosome 2B - were detected near the genome-wide significance threshold. While these two markers did not surpass Bonferroni-threshold, their proximity to it suggests a potential involvement in crown rot resistance. These regions may contain genes with smaller effect size that contribute to the resistance and warrant further investigations.

In this study, we evaluated 100 exotic wild *Fragaria* accessions from diverse geographical origins for resistance to Phytophthora crown rot. The introgression of favourable alleles from this exotic gene pool, particularly from subspecies that are underutilised in breeding, such as the North American *F. chiloensis* ssp. *pacifica* and ssp. *lucida*,* F. virginiana* ssp. *platypetala* [27] and *F. virginiana* ssp. *grayana* [103], holds considerable promise for broadening the resistance base of existing strawberry cultivars. Our analysis also revealed that the genomic region surrounding the significant SNP marker harbours a high density of defence-related genes, indicating its potential for fine-mapping and functional characterisation. Within this interval, the NLR cluster, together with adjacent RLKs and Ca²⁺ signalling genes, represents the most compelling target for functional follow-up, as these gene classes provide a direct mechanistic link to pathogen recognition and early immune signal transduction. While both GWAS models used in this study converged towards the same region as associated with disease resistance, further validation through phenotyping of larger sample populations and the use of alternative genotyping platforms would be useful for further studies. Additional validation in segregating F1 populations derived from crosses between resistant wild accessions and susceptible cultivars, together with fine-mapping and flanking markers, will be required to confirm the marker’s efficacy in an octoploid breeding background. In light of the methyl bromide phase-out, the rising prevalence of fungicide resistant pathogens, and the lack of fully resistant commercial cultivars, genome-informed, marker-assisted selection represents a practical strategy to enhance quantitative resistance and support sustainable genetic improvement of the strawberry plant.

## Supplementary Information

Supplementary Material 1.

Supplementary Material 2.

Supplementary Material 3.

Supplementary Material 4.

## Data Availability

All data supporting the findings of this study are available within the paper and its Supplementary Information.
