# Supplementary material for: Genome-wide association study of exotic Fragaria germplasm accessions for resistance to Phytophthora crown rot in strawberry
Source: BMC Plant Biol. 2026 Jan 23;26:324. doi: 10.1186/s12870-026-08186-6 (PMC12911263; doi:10.1186/s12870-026-08186-6)
Supplement: Supplementary file 1 — Supplementary Material 1. [file 12870_2026_8186_MOESM1_ESM.docx]

**Table S1** SNP categories according to their performance in the Axiom™ FanaSNP 50k Genotyping Array.

| **SNPolisher category ^a^** | **Number of SNPs** | **% of total SNPs** |
| --- | --- | --- |
| Poly High Resolution (PHR) | 27,721 | 56.0 |
| No Minor Homozygote (NMH) | 3,974 | 8.0 |
| Mono High Resolution (MHR) | 466 | 0.9 |
| Call Rate Below Threshold (CRBT) | 4,497 | 9.1 |
| Off-Target Variant (OTV) | 3,368 | 6.8 |
| Other | 9,457 | 19.1 |
| Total | 49,483 | 100 |

^a^ Distribution of SNP markers from 100 wild *Fragaria* accessions classified into six categories based on their clustering performance.

**Table S2** Distribution of 15,633 SNP markers across 28 *Fragaria* chromosomes, with their average distance and percentage per sub-genome.

| **Chromosome** | **Chromosome size (Mb)** | **Number of SNPs** | **SNP density (kb per SNP) ^a^** | **Markers (%) per subgenome** | **LD decay distance (Mb)** | **Half LD decay (r^2^)** |
| --- | --- | --- | --- | --- | --- | --- |
| 1A | 22.9 | 577 | 39.6 | 28.2 | 2.76 | 0.23 |
| 1B | 25.9 | 473 | 54.8 | 23.1 | 0.84 | 0.23 |
| 1C | 25.7 | 577 | 44.6 | 28.2 | 2.51 | 0.24 |
| 1D | 26.3 | 421 | 62.4 | 20.6 | 0.93 | 0.24 |
| 2A | 27.5 | 591 | 46.5 | 27.1 | 3.02 | 0.23 |
| 2B | 27.4 | 500 | 54.8 | 22.9 | 1.03 | 0.23 |
| 2C | 27.2 | 557 | 48.9 | 25.5 | 1.12 | 0.23 |
| 2D | 28.1 | 533 | 52.7 | 24.4 | 2.00 | 0.23 |
| 3A | 31.4 | 421 | 74.6 | 17.8 | 0.58 | 0.23 |
| 3B | 31.3 | 686 | 45.6 | 29.0 | 1.61 | 0.24 |
| 3C | 31.1 | 610 | 51.0 | 25.8 | 1.76 | 0.23 |
| 3D | 30.2 | 646 | 46.7 | 27.3 | 1.69 | 0.23 |
| 4A | 26.7 | 593 | 45.0 | 30.7 | 3.98 | 0.23 |
| 4B | 30.4 | 485 | 62.8 | 25.1 | 2.40 | 0.23 |
| 4C | 26.8 | 500 | 53.6 | 25.9 | 1.60 | 0.23 |
| 4D | 24.9 | 351 | 71.0 | 18.2 | 0.71 | 0.23 |
| 5A | 26.4 | 490 | 53.9 | 22.1 | 1.94 | 0.23 |
| 5B | 29.2 | 644 | 45.3 | 29.0 | 3.20 | 0.23 |
| 5C | 27.0 | 582 | 46.5 | 26.3 | 3.54 | 0.24 |
| 5D | 26.1 | 501 | 52.1 | 22.6 | 0.61 | 0.23 |
| 6A | 34.4 | 688 | 50.1 | 23.5 | 1.05 | 0.23 |
| 6B | 36.0 | 693 | 51.9 | 23.7 | 0.55 | 0.23 |
| 6C | 35.3 | 725 | 48.6 | 24.8 | 2.35 | 0.24 |
| 6D | 32.9 | 819 | 40.2 | 28.0 | 1.95 | 0.23 |
| 7A | 22.6 | 518 | 43.5 | 26.3 | 2.54 | 0.23 |
| 7B | 23.2 | 517 | 44.9 | 26.2 | 0.99 | 0.23 |
| 7C | 22.9 | 459 | 49.8 | 23.3 | 1.67 | 0.24 |
| 7D | 22.6 | 476 | 47.5 | 24.2 | 4.31 | 0.23 |
| **Total** | 782.4 | 15,633 |  |  |  |  |
| **Average** | 27.9 | 558.3 | 51.0 |  | 1.90 | 0.23 |

^a^ SNP density is the mean physical distance between two adjacent marker loci.


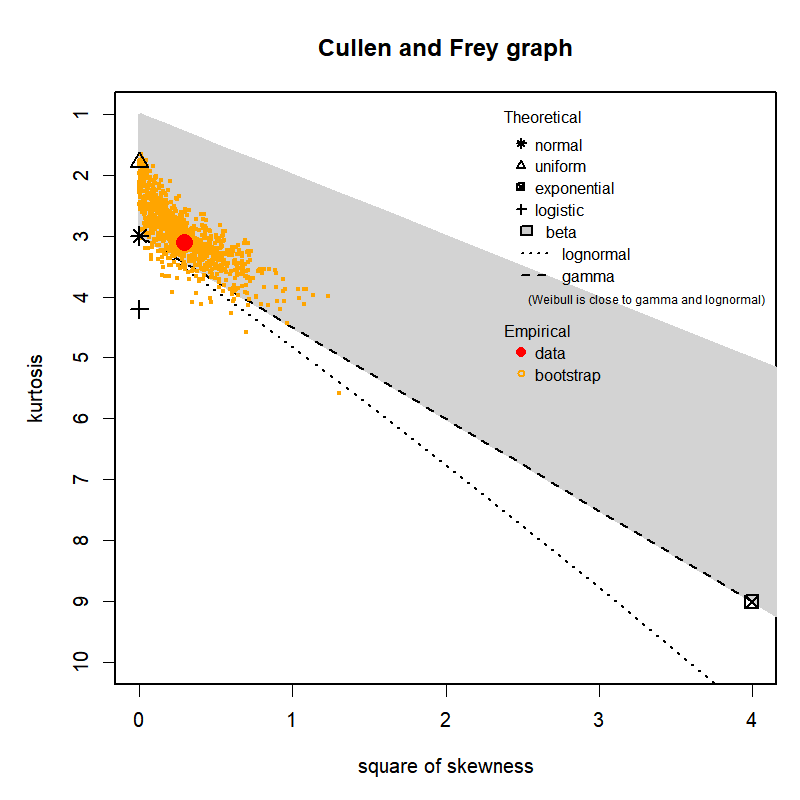
**Fig. S1** Cullen and Frey skewness-kurtosis plot comparing the observed phenotypic distribution (red dot) against theoretical distributions, based on 1000 bootstrap replicates. The observed phenotypic distribution lies close to the reference point for a normal distribution, indicating approximate normality.


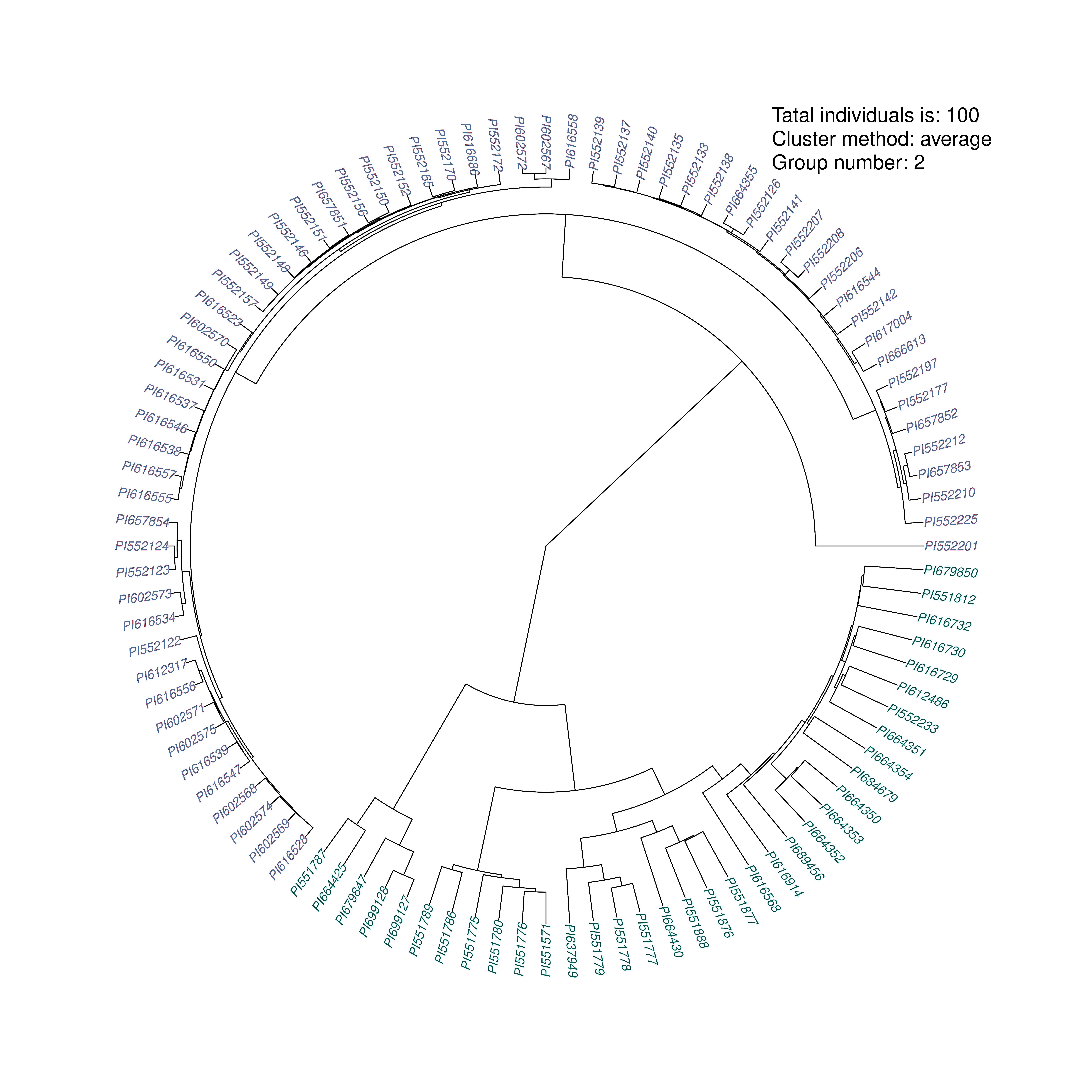
**Fig. S2** Phylogenetic tree of 100 wild Fragaria accessions based on 2,849 linkage disequilibrium (LD) pruned SNP markers. The tree was generated using the neighbour-joining (NJ) method implemented in GAPIT 3.


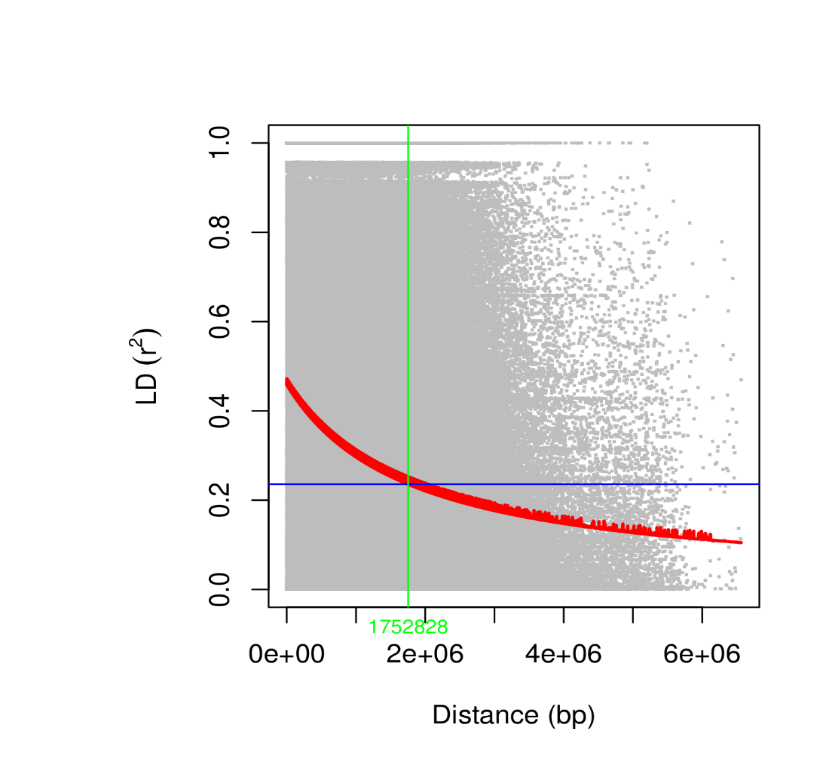
 **Fig. S3** Genome wide linkage disequilibrium (LD) decay plot. Pairwise LD values (r^2^) are plotted against the corresponding physical distance (bp) between SNP pairs. The horizontal blue line indicates the average half-decay threshold (r² = 0.23), representing the distance at which LD decays to half of its maximum value. The vertical green line marks the estimated average physical distance (1.75 Mb) where this half-decay occurs.


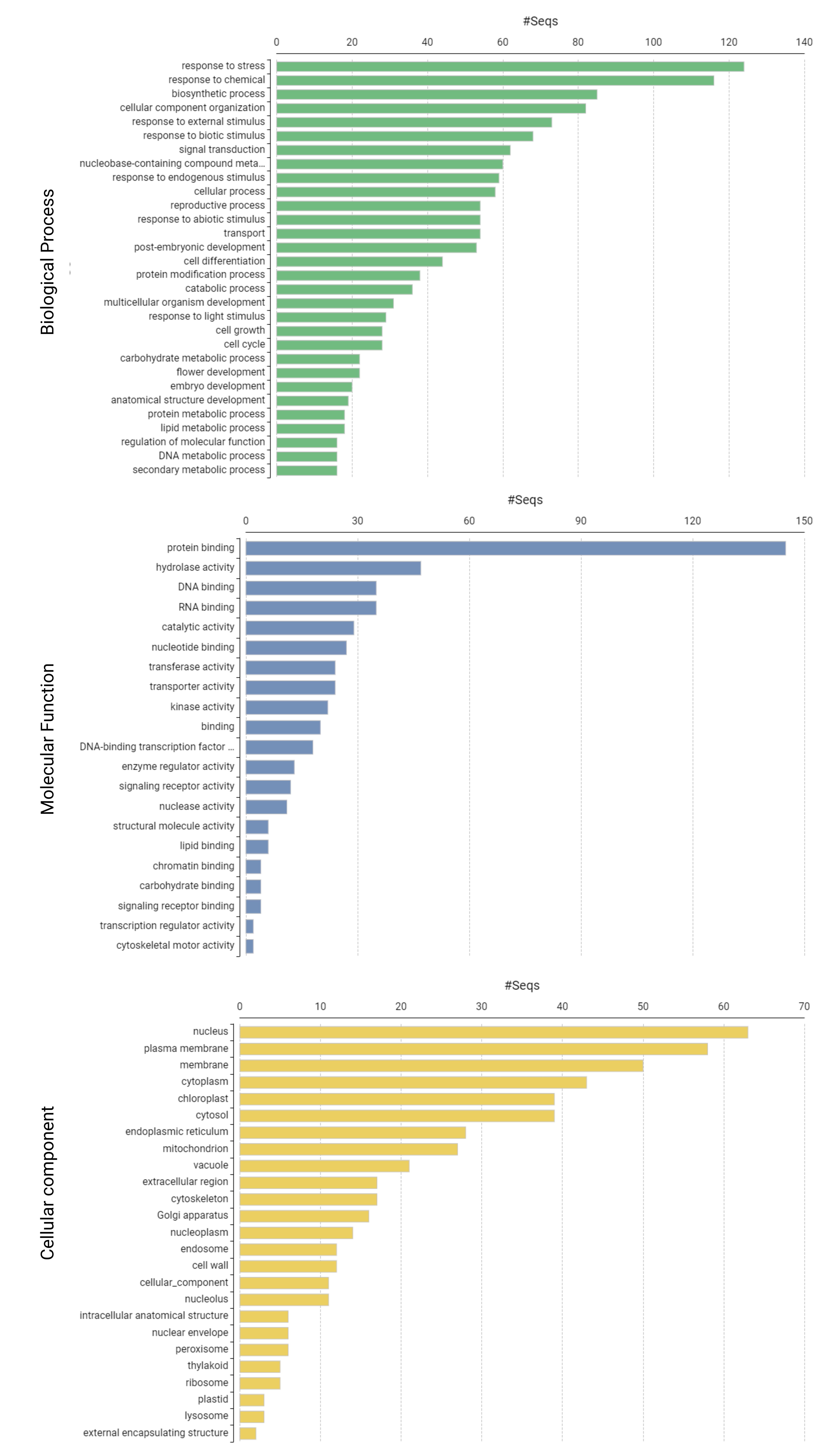
**Fig. S4** Gene Ontology (GO) term functional classification of genes within the 2 Mb genomic region associated with *Phytophthora* crown rot resistance. Histograms show the number of sequences assigned to specific GO categories: Biological Processes, Molecular Functions, and Cellular Components, as determined using Blast2GO.
